# Identification of mitochondrial respiratory chain signature for predicting prognosis and immunotherapy response in stomach adenocarcinoma

**DOI:** 10.1186/s12935-023-02913-x

**Published:** 2023-04-16

**Authors:** Jing Yang, Feifan Jin, Huanjuan Li, Yuhuan Shen, Weilin Shi, Lina Wang, Lei Zhong, Gongqiang Wu, Qiaoliang Wu, Yanchun Li

**Affiliations:** 1 Laboratory Medicine Center, Department of Laboratory Medicine, Affiliated People’s Hospital, Zhejiang Provincial People’s Hospital, Hangzhou Medical College, Hangzhou, Zhejiang 310014 China; 2 Department of Central Laboratory, Affiliated Hangzhou first people’s Hospital, Zhejiang University School of Medicine, Hangzhou, Zhejiang 310006 China; 3 Center for Plastic & Reconstructive Surgery, Department of Stomatology, Affiliated People’s Hospital, Zhejiang Provincial People’s Hospital, Hangzhou Medical College, Hangzhou, Zhejiang 310014 China; 4 Department of Medicine, Taizhou Luqiao District Second People’s Hospital, Taizhou, Zhejiang 318058 China; 5 Department of Clinical Laboratory, Tongxiang Hospital of Traditional Chinese Medicine, Jiaxing, Zhejiang 314599 China; 6 Department of Hematology, Dongyang People’s Hospital, Dongyang Hospital Affiliated to Wenzhou Medical University, Dongyang, Zhejiang 322100 China; 7 Department of Hematology, Jiashan first people’s Hospital, Jiaxing, Zhejiang 314199 China

**Keywords:** Stomach adenocarcinoma, Mitochondrial complexes, Tumor microenvironment, Microsatellite instability, Prognosis

## Abstract

Stomach adenocarcinoma (STAD) is the third leading cause of cancer-related deaths and the fifth most prevalent malignancy worldwide. Mitochondrial respiratory chain complexes play a crucial role in STAD pathogenesis. However, how mitochondrial respiratory chain complex genes (MRCCGs) affect the prognosis and tumor microenvironment in STAD remains unclear. In this study, we systematically analyzed genetic alterations and copy number variations of different expression densities of MRCCGs, based on 806 samples from two independent STAD cohorts. Then we employed the unsupervised clustering method to classify the samples into three expression patterns based on the prognostic MRCCG expressions, and found that they were involved in different biological pathways and correlated with the clinicopathological characteristics, immune cell infiltration, and prognosis of STAD. Subsequently, we conducted a univariate Cox regression analysis to identify the prognostic value of 1175 subtype-related differentially expressed genes (DEGs) and screened out 555 prognostic-related genes. Principal component analysis was performed and developed the MG score system to quantify MRCCG patterns of STAD. The prognostic significance of MG Score was validated in three cohorts. The low MG score group, characterized by increased microsatellite instability-high (MSI-H), tumor mutation burden (TMB), PD-L1 expression, had a better prognosis. Interestingly, we demonstrated MRCCG patterns score could predict the sensitivity to ferroptosis inducing therapy. Our comprehensive analysis of MRCCGs in STAD demonstrated their potential roles in the tumor-immune-stromal microenvironment, clinicopathological features, and prognosis. Our findings highlight that MRCCGs may provide a new understanding of immunotherapy strategies for gastric cancer and provide a new perspective on the development of personalized immune therapeutic strategies for patients with STAD.

**Supplementary Information:**

The online version contains supplementary material available at 10.1186/s12935-023-02913-x.

## Introduction

Stomach adenocarcinoma (STAD) is one of the most common gastrointestinal cancers, which ranks fifth in cancer incidence and third in cancer mortality worldwide due to its rapid progress to advanced stages [1, 2]. Many factors can facilitate its initiation and progression, including the environment and genetics [3]. The incidence of STAD increases progressively with age, and most patients are diagnosed at an advanced stage [4]. Although great advances have been made in endoscopic and surgical therapies, chemotherapy, and systemic treatments, a myriad of STAD patients who are diagnosed in the advanced stage do not benefit from it [5]. Therefore, exploring early diagnostic tools and effective treatment drugs are the most useful strategies for improving the prognosis of STAD patients. Recently, immune checkpoint inhibitors (ICIs) and other targeted medicine have been used for STAD treatment; however, their response effect is limited to some patients [6]. Multiple researchers have shown genomics has become an indispensable tool for STAD treatment which is expected to be further developed in the future [6, 7].

Warburg proposed that tumors prefer aerobic glycolysis due to a defect in mitochondrial oxidative phosphorylation (OXPHOS) [8]. OXPHOS is undertaken by mitochondrial respiratory chain complexes (MRCCs), which are formed by four multi-subunit complexes (complex I–IV; CI, NADH: ubiquinone oxidoreductase; CII, succinate: ubiquinone oxidoreductase; CIII, cytochrome bc1 complex; and CIV, cytochrome c oxidase), two mobile electron carriers, and an ATP synthase (also called CV) [9, 10]. MRCCs are located in the inner mitochondrial membrane and play a significant role in energy conversion [9]. Previous studies showed that the MRCCs plays a vital role in cancer metabolism [11–14]. Gastric carcinomas exhibit a higher percentage of OXPHOS enzyme defects than adjacent control tissues [15, 16]. Moreover, Zhao et al. reported that dizen-1‐ium‐1,2‐diolate (JS‐K) targets mitochondrial CI and CIV to exert a reactive oxygen species (ROS)-dependent anti-cancer function in gastric cancer [17]. Single or mitochondrial respiratory chain complexes play crucial roles in the pathogenesis of gastric carcinomas. However, the whole landscape of MRCCGs in prognostic roles of STAD remains largely unexplored.

Tumor immune microenvironment (TME), including endothelial cells, immune and inflammatory cells such as lymphocytes and macrophages; and stromal cells such as fibroblasts, adipocytes, and pericytes; has emerged as an important determinant of tumor progression and therapeutic response [18]. Among them, immune cells were recognized as emerging hallmarks of cancer [19]. Thus a series of immunotherapy approaches were developed and clinically applied by harnessing the immune cells within or outside the TME to specially recognize and attack the cancer cells [20]. In recent years, TME has emerged as a potential therapeutic target in STAD, including targeting Tumor-Associated Macrophages (TAM), tumor-infiltrating lymphocytes (TILs), Cancer-Associated Fibroblasts (CAFs), and Mesenchymal Stem Cells (MSCs) [21]. Hence, understanding the molecular and cellular biology of TME will conduce to the discovery of promising new therapeutic approaches in the treatment of STAD.

OXPHOS appears of significance in the TME. It provides the required energy for cancer cells and stromal cells to differentiate into OXPHOS-dependent cancer stem cells with primary or acquired resistance against chemotherapy or tyrosine kinase inhibitors [22, 23]. Recent scientific evidence has demonstrated that inducing mitochondrial oxidative stress in cancer-associated fibroblasts, a major cellular component of the tumor stroma and generated from various TME cell types, is a key event in cancers via TME formation [24]. With the development of precision medicine, OXPHOS analysis provides evidence for the clinical application of chemotherapy options in uterine corpus endometrial carcinoma [25]. However, to the best of our knowledge, OXPHOS combined with TME has not been included in STAD prognostic models in previous studies. Therefore, a comprehensive understanding of the characteristics of TME cell infiltration mediated by multiple MRCCGs may be helpful to better understand the underlying mechanisms of STAD tumorigenesis and predict the response to immunotherapy.

This study systematically evaluated the expression profiles of MRCCGs from The Cancer Genome Atlas (TCGA) cohort and Gene-Expression Omnibus (GEO) datasets and obtained a comprehensive overview of the intratumoral immune landscape using CIBERSORT. STAD patients were stratified into three discrete patterns according to MRCCG expression levels. We further evaluated the relationship between different MRCCG patterns and immune cell infiltration characteristics. Patients were then classified into three gene subtypes based on the differentially expressed genes (DEGs) identified in the three MRCCG patterns. More importantly, we established an MG score to predict prognosis and characterize the immune landscape of STAD, which accurately predicted patients’ response to immunotherapy. Collectively, our study suggests that MRCCGs play a crucial role in the formation of the TME and could be a guide for immunotherapy and ferroptosis based therapy for patients with STAD.

## Materials and methods

### Stomach adenocarcinoma data sources

Data sources, including RNA sequencing data (fragments per kilobase million, FPKM), genome mutation data, relevant prognostic and corresponding clinical information of stomach adenocarcinoma (STAD) patients were downloaded from TCGA (https://tcga-data.nci.nih.gov/tcga/) databases (373 samples) and GSE84437 (https://www.ncbi.nlm.nih.gov/geo/) dataset (433 samples). FPKM values of the RNA data were converted to transcripts per kilobase million (TPM) by employing FPKM function of the “limma” package in R. The inclusion criteria of STAD samples were as follows: (i) gene expression profiling of STAD samples was available in the dataset; (ii) complete clinical data of STAD patients, including gender, age, TNM stage, and overall survival. Compliant data sets were subjected to copy number variation (CNV) analysis. The plot of MRCCGs copy number changes in the chromosome was drawn using the “Rcircos” package.

### Consensus clustering analysis of 24 MRCCGs

96 MRCCGs were retrieved from wiki pathways (https://www.wikipathways.org/). The full details of these genes are shown in Table S1. The differential analysis found that 24 MRCCGs were significantly different between STAD tissues and normal tissues. The information on these genes was depicted in Table S2. These 24 MRCCGs were used to screen distinct patterns of STAD in our study. Unsupervised cluster analysis in the “ConsensuClusterPlus” package was applied to classify patients into distinct gene patterns according to the expression of 24 MRCCGs.

### Relationship between MRCCGs patterns with the clinical features and prognosis of STAD

To characterize the clinical features of the three patterns identified by consensus clustering analysis, we compared the relationships between molecular patterns, clinicopathological characteristics, and prognosis. The patient’s clinicopathological characteristics included age, gender, TNM stage, and survival status. The Cox regression model was used to evaluate the survival prognostic differences of three MRCCG patterns. Furthermore, the prognosis among different patterns was assessed using Kaplan–Meier curves generated by the “survival” and “survminer” R packages.

### Gene set variation analysis (GSVA) and gene enrichment function annotation

To investigate the differences in 24 MRCCGs in biological processes, gene set variation analysis (GSVA) was performed with the hallmark gene set (c2. cp.kegg.v7.2) derived from the MSigDB database [26]. The cluster profile R Package was used to functionally study the difference in the activities of MRCCG patterns [27]. The gene ontology (GO) function annotations and kyoto encyclopedia of genes and genomes (KEGG) of MRCCGs were analyzed using the “clusterProfiler” package. Adjusted p-value < 0.05 and FDR < 0.01 were considered statistically significant.

### The TME cell infiltrating characteristics analysis of the different MRCCG patterns

To observe the difference between the MRCCG patterns and TME infiltrating immune cells, we used the CIBERSORT algorithm (https://cibersort.stanford.edu/) to calculate the fractions of 23 human immune cells subsets. Furthermore, the levels of immune cell infiltration in the STAD were also examined using a single-sample gene set enrichment analysis (ssGSEA) algorithm [28].

### Screening of DEGs among different MRCCG patterns and functional annotation

The R package “limma” was used to screen the MRCCG pattern-related DEGs. The gene with an adjusted p-value of < 0.001 was identified as significant DEGs. To further explore the potential functions of MRCCGs pattern-related DEGs and identify the related gene functions, the gene ontology (GO) and Kyoto Encyclopedia of Genes and Genomes (KEGG) enrichment analysis of DEGs were performed using the clusterProfiler R package. FDR < 0.01 was considered statistically significant.

### Construction of MRCCGs signature

We constructed a scoring system termed as MG score, which was calculated to quantify the different MRCCG patterns of the individual STAD. First, we executed the prognostic analysis for all MRCCG pattern-related DEGs in the signature by using univariate Cox regression analysis. Second, the patients were classified into three different gene subtype groups (gene cluster 1, gene cluster 2, and gene cluster 3) for deeper analysis using a consensus clustering algorithm based on the expression of overlapped genes. Finally, PCA with the “ggplot2” R package was employed to construct MG score. Principal components 1 and 2 were selected as signature scores. MG score = (PC1i + PC2i). where is the expression of subtype-specific genes. Based on the median risk score, patients were divided into the high MG score group and low MG score group, each of which was subjected to Kaplan–Meier survival analysis (log-rank tests, p < 0.001).

### Mutation and immunotherapeutic susceptibility analysis

To study the somatic mutations of STAD patients between high-MG and low-MG score groups, we calculated the tumor mutation burden (TMB) score for each patient with STAD using the “maftools” R package. We used the Wilcoxon test to explore differences in the therapeutic effects of immunotherapeutics in patients with high MG scores or low MG scores.

### Cell culture

Human gastric cancer cells, BGC823, were preserved and passaged in our laboratory and cultured using DMEM medium (Hyclone, Logan, UT, USA) supplemented with 10% fetal bovine serum (Gibco, Grand Island, NY, USA), penicillin (100 U/mL) and streptomycin (100 µg/mL). All cells were cultured at 37 °C and the plates were placed in a CO_2_ incubator in which the gas composition was 95 vol% air and 5 vol% CO_2_.

### CCK8 assay

Cell viability was measured by Cell Counting Kit-8 assay (CCK-8) according to the manufacturer’s instructions. Cells were seeded in 96-well culture plates (Nest, Biotechnology) at a density of 2 × 10^4^ cells /well. A series of agents including mitochondrial respiration inhibitors (antimycin A and TTFA) and mitochondrial energy metabolic substrate (Dimethyl fumarate) was administered in combination with erastin to cells for 36 h at 37 °C. Then the supernatant was replaced with CCK8-containing medium for additional 2 h and assayed for cell viability by measuring the absorbance at 450 nm. Each experiment was repeated three times.

### Fluorescent probes staining

BGC823 cells (700,000 per well) were placed in 6-well plates with indicated treatment. The culture solution was discarded, then cells were stained with BODIPY (4 µM). DAPI was used for nuclear staining. After staining in the incubator for 30 min, Cells were washed three times with PBS to remove excess BODIPY and subsequently viewed and captured under a confocal microscope.

### Statistical Analysis

T-test was used to perform differential analysis between STAD and corresponding normal tissues. Correlation coefficients between the TME-infiltrating immune cells and the expression of MRCCGs were calculated by Spearman and differential expression analyses. Analysis of Variance (ANOVA) and the Kruskal-Wallis test were used to compare differences between the three groups. Univariate regression analyses were utilized to calculate the hazard ratios (HR) for MRCCGs and MRCCGs -related genes. Kaplan–Meier method was utilized to perform survival curves for the prognostic analysis and the log-rank test was used to determine the significance of the differences. The differences in immune subtypes proportion between high MG score and low MG score groups were calculated by chi-square test. All statistical analyses were performed with R version 4.1.0. In the *vitro* experiment, differences between the two groups were assessed with Student’s t-test and comparisons among multi groups were evaluated by the analysis of variance (ANOVA) using GraphPad Prism version 5.0. The value of *P* < 0.05 was considered statistically significant.

## Results

### Genetic and transcriptional alterations of differential MRCCGs in STAD

**Fig. 1:**
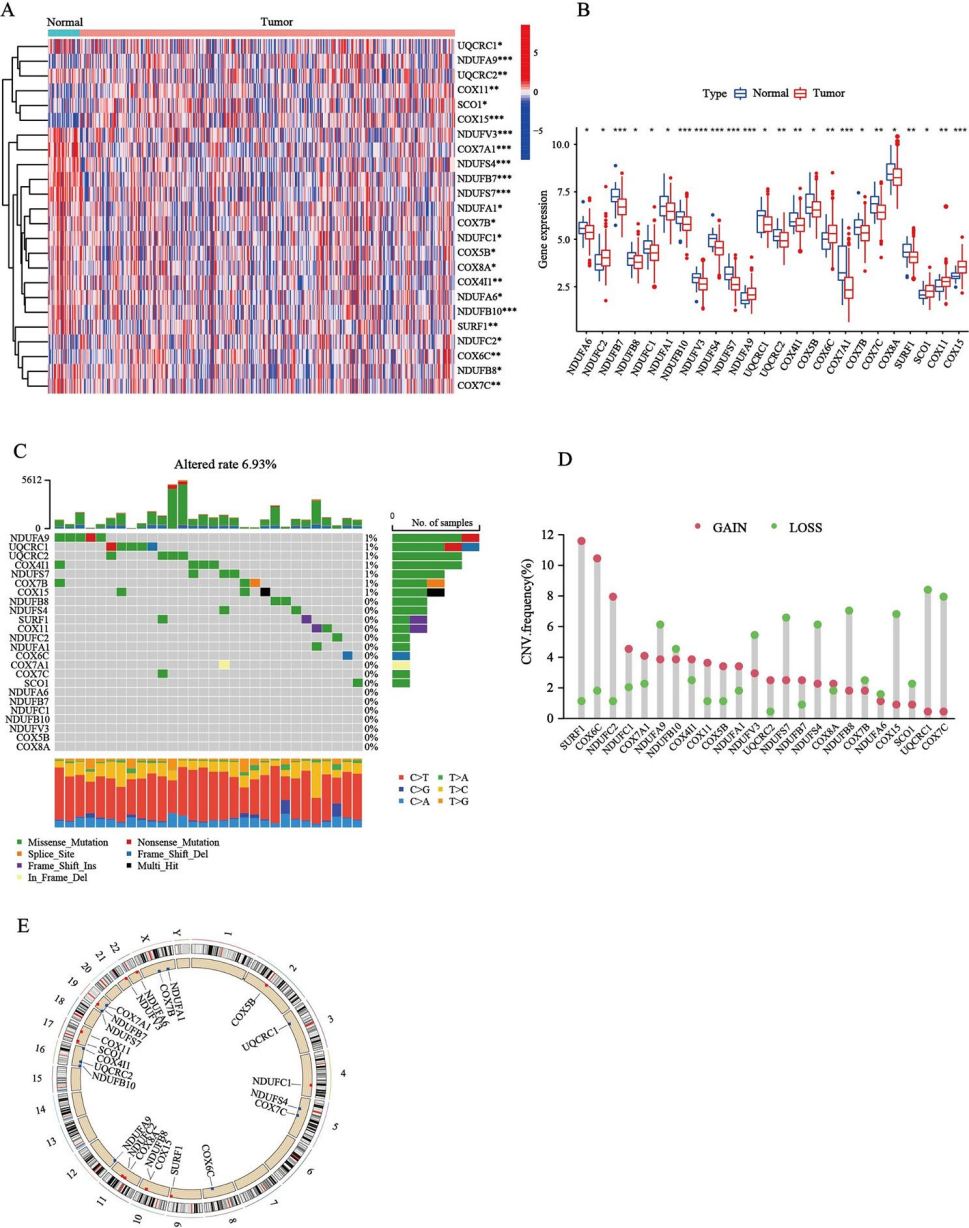
**Genetic landscape and alterations of MRCCGs in STAD.** **(A)** The heatmap of the expression distributions of the 24 differential MRCCGs between normal and tumor samples. Red or blue represents upregulation and downregulation, respectively. **(B)** Box plot displaying the expression distributions of MRCCGs crosstalk between normal and tumor samples. Red or blue dots represent tumor and normal samples, respectively. **(C)** Mutation frequency of the 24 different expressed mitochondrial complex genes of STAD patients in the TCGA-STAD and GSE84437 cohorts. **(D)** Frequencies of CNV gain, loss, and non-CNV among MRCCGs. The abscissa was the MRCCGs, and the ordinate was the mutation frequency. Red represents an increase in copy number, and green represents the loss of copy number. **(E)** Locations of CNV alterations of MRCCGs on 24 chromosomes. The *** represents p < 0.001, ** represents p < 0.01, * represents p < 0.05

This study included 96 MRCCGs, including 41 CI, 4 CII, 9 CIII, 23 CIV, and 19 CV genes. Among them, 24 MRCCGs with different expressions were selected for further differential analysis. The heat map in Fig. 1A depicts the differences of 24 MRCCGs between STAD and normal tissues. Figure 1B shows the 24 differential MRCCGs, of which only 6 genes were upregulated and 18 genes were downregulated in the STAD group. To determine the genetic alterations in RNA levels of MRCCGs in STAD, we assessed the prevalence of mutations in these 24 differently expressed MRCCGs. We found that the overall mutation rate of all genes is relatively low in the STAD genome (Fig. 1C). Next, we explored the somatic copy number alterations in these MRCCGs and discovered prevalent copy number alterations in all 24 MRCCGs. Among them, *SURF1*, *COX6C*, *NDUFC2*, and *NDUFC1* had widespread copy number variation (CNV) gain, whereas *UQCRC1*, *COX7C*, *COX15*, and *NDUFB8* showed CNV loss (Fig. 1D). Consistently, MRCCGs with a higher mutation rate (*NDUFA9*, *UQCRC1*, *UQCRC2*, *COX4I1*, *NDUFS7*, *COX7B*, or *COX15*) were found to have a higher frequency of CNV loss than CNV gain. Figure 1E depicts a copy number circle diagram, which shows the CNV mutation locations of each MRCCG on the chromosomes.

We further analyzed the OS (overall survival) of patients with STAD based on the expression of the MRCCGs. The patients were divided into high- and low-expression groups according to the median expression of these genes. The results showed that most MRCCGs had a strong correlation with the survival outcome of STAD patients (Fig. S1). Collectively, the results of our analysis showed a significant difference in the genetic landscape of MRCCGs, and its expression level was correlated with the OS of STAD patients, indicating the latent function of MRCCGs in STAD oncogenesis.

### Identification of MRCCGs patterns in STAD

**Fig. 2:**
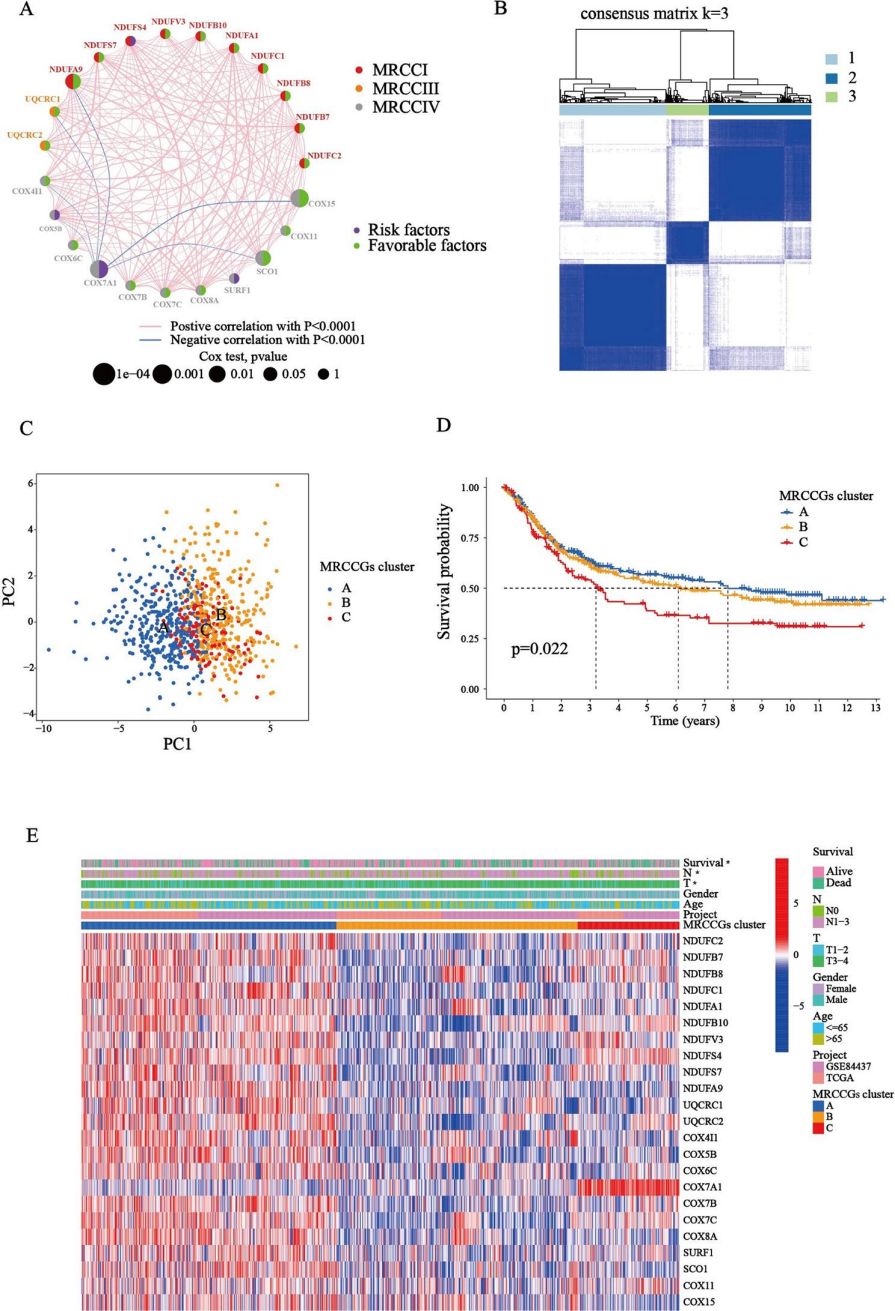
**MRCCGs patterns and clinicopathological characteristics** **(A)** Interactions among MRCCGs in STAD. The line connecting the MRCCGs represents their interactions, with the line thickness indicating the strength of the association between MRCCGs. The size of each circle indicates the different significance of each gene. Blue and pink represent negative and positive correlations, respectively. Favorable factors are indicated in green, and risk factors are indicated in purple. **(B)** Consensus matrix heatmap defining three clusters (k = 3) and their correlation area. **(C)** PCA analysis shows a remarkable difference in transcriptomes among the three patterns. **(D)** Kaplan–Meier curves of the overall survival in the TCGA-STAD and GSE84437 cohorts. **(E)** Heat map showing differences in clinicopathologic features and expression levels of MRCCGs between the three distinct patterns. STAD, stomach adenocarcinoma; MRCCGs, mitochondrial respiratory chain complexes genes; PCA, principal components analysis

To fully understand the gene expression patterns of MRCCGs in STAD, 806 patients from two cohorts (TCGA-STAD and GSE84437) were integrated into our study for further analysis. The prognosis network diagram showed that most MRCCGs were positively correlated, with only negative correlations between *COX7A1* and *UQCRC1*, *COX5B*, *COX4I1*, *NDUFA9*, *SCO1*, and *COX15* (Fig. 2A). To further explore the expression characteristics of MRCCGs in STAD, we used a consensus clustering algorithm to categorize patients with STAD based on the expression profiles of MRCCGs. Notably, our results showed that k = 3 appeared to be the optimal selection for sorting the entire cohort into MRCCG patterns A, B, and C (Fig. 2B, Fig. S2). The PCA revealed significant differences in the transcriptome profiles of the three patterns (Fig. 2C). Survival analysis of the MRCCG patterns showed a longer OS in patients with pattern A than patients with the other two MRCCG patterns (log-rank test, *p* = 0.022; Fig. 2D). Furthermore, a comparison of the clinicopathological features of the different patterns demonstrated remarkable differences in MRCCG expression and clinicopathological characteristics (Fig. 2E). Cluster A was enriched with the highest MRCCG expression, whereas cluster B displayed the lowest MRCCG expression.

### Characteristics of TME and biological processes under different MRCCG patterns

**Fig. 3:**
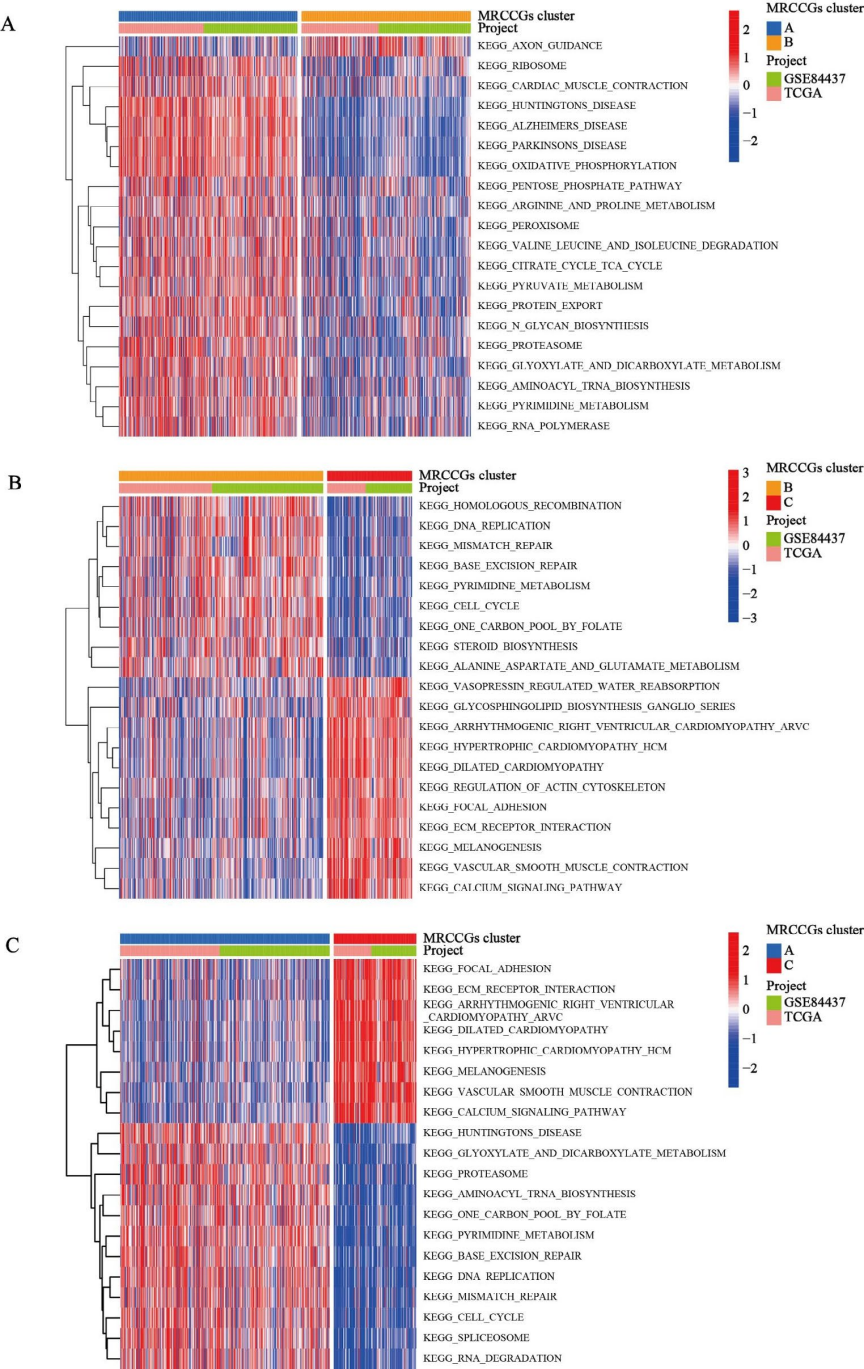
**Biological characteristics of MRCCG patterns** **(A)** GSVA analyzed the differences between functional pathways in MRCCGs pattern A and B. Blue represents the MRCCGs pattern A, and orange represents the MRCCGs pattern B. **(B)** GSVA analyzed the differences between functional pathways in MRCCGs pattern B and C. Orange represents the MRCCGs pattern A, and red represents the MRCCGs pattern C. **(C)** GSVA analyzed the differences between functional pathways in MRCCGs pattern A and C. Blue represents the MRCCGs pattern B, and red represents the MRCCGs pattern C. The *** represents p < 0.001, ** represents p < 0.01 and ns represents no significance. GSVA, gene set variation analysis

**Fig. 4:**
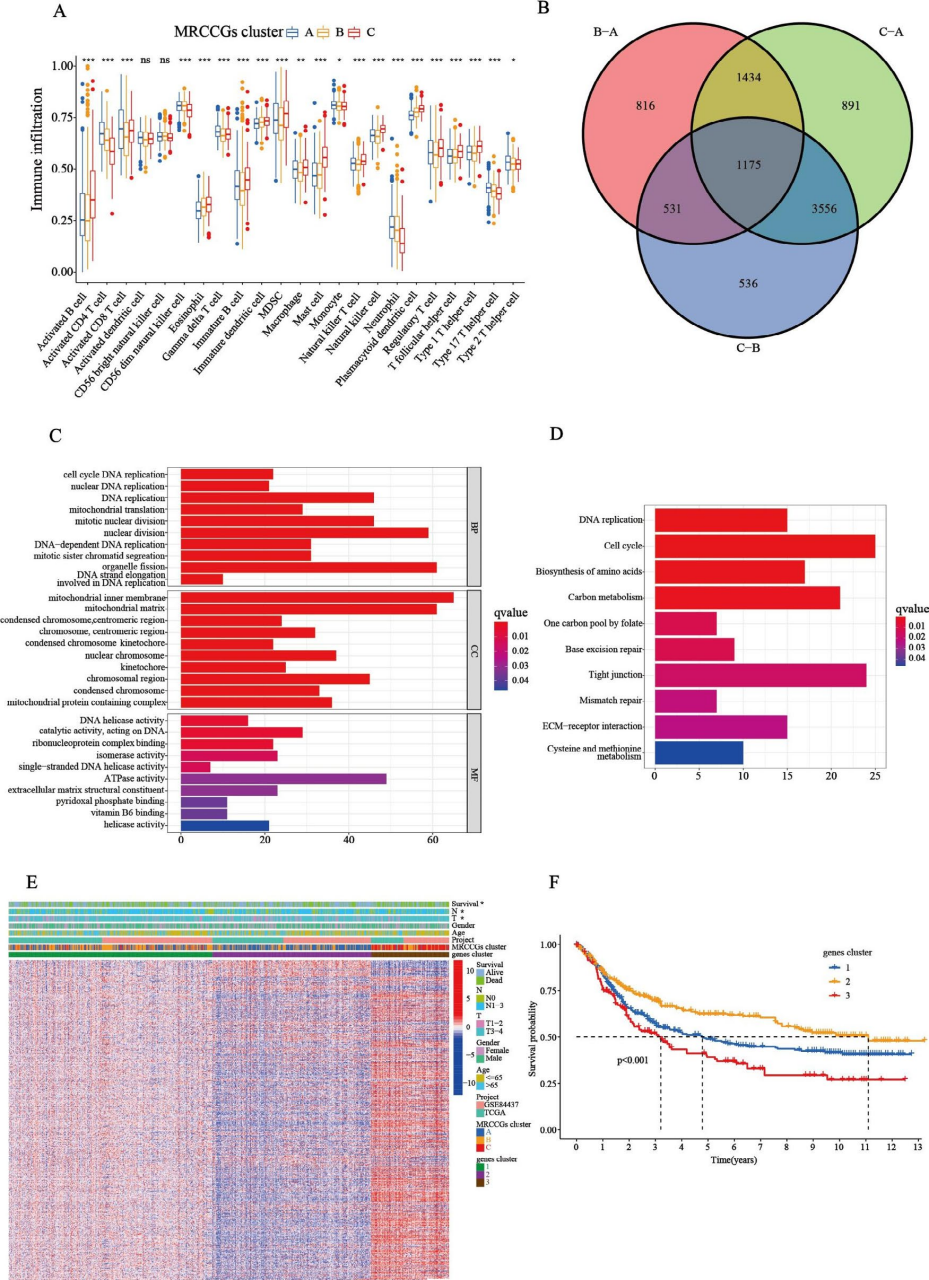
**Identification of gene subtypes based on DEGs.** **(A)** The differential expression analysis of 23 immune cells among three MRCCG patterns. **(B)** The number of unique and shared DEGs from different comparisons is revealed in a Venn diagram. **(C-D)** GO and KEGG enrichment analyses of DEGs among three MRCCG patterns. **(E)** Relationships between clinicopathologic features and the three gene subtypes. **(F)** Kaplan–Meier curves of the three gene subtypes (log-rank tests, p < 0.001). Blue represents gene cluster 1, orange represents gene cluster 2, and red represents gene cluster 3. DEGs, differentially expressed genes; GO, Gene Ontology; KEGG, Kyoto Encyclopedia of Genes and Genomes

GSVA was used to investigate the differences in the biological functions of different MRCCG patterns. As shown in Fig. 3A–C, we observed differences in the functional pathways between different MRCCG patterns. Pattern A was significantly enriched in central nervous system diseases, such as Huntington’s disease and Parkinson’s disease, and energy metabolism pathways, such as oxidative phosphorylation. Pattern B was mainly concentrated in the cell cycle, DNA replication, mismatch repair, and base excision repair pathways. Pattern C was associated with focal adhesion, melanogenesis, vascular smooth muscle contraction, and ECM receptor interaction pathways. To investigate the role of MRCCGs in the TME of STAD, we assessed the correlations between the three patterns and 23 human immune cell subsets using the CIBERSORT algorithm. The three MRCCG patterns showed significantly different infiltration characteristics of the TME cells (Fig. 4A). The infiltration levels of activated CD4^+^ T cells, CD8^+^ T cells, gamma delta T cells, neutrophils, and type 17 helper T cells were significantly higher in pattern A than those in patterns B and C, while activated B cells, plasmacytoid, follicular helper T cells, type 1 helper T cells, natural killer (NK) T cells, NK cells, macrophages, mast cells, immature dendritic cells, eosinophils, MDSCs, and regulatory T cells (Tregs) were significantly higher in pattern C. These results suggest that the three categorized MRCCG patterns have significantly different biological characteristics and immune infiltration patterns, which could discriminate the prognosis of STAD patients.

### Identification of gene subtypes based on DEGs

**Fig. 5:**
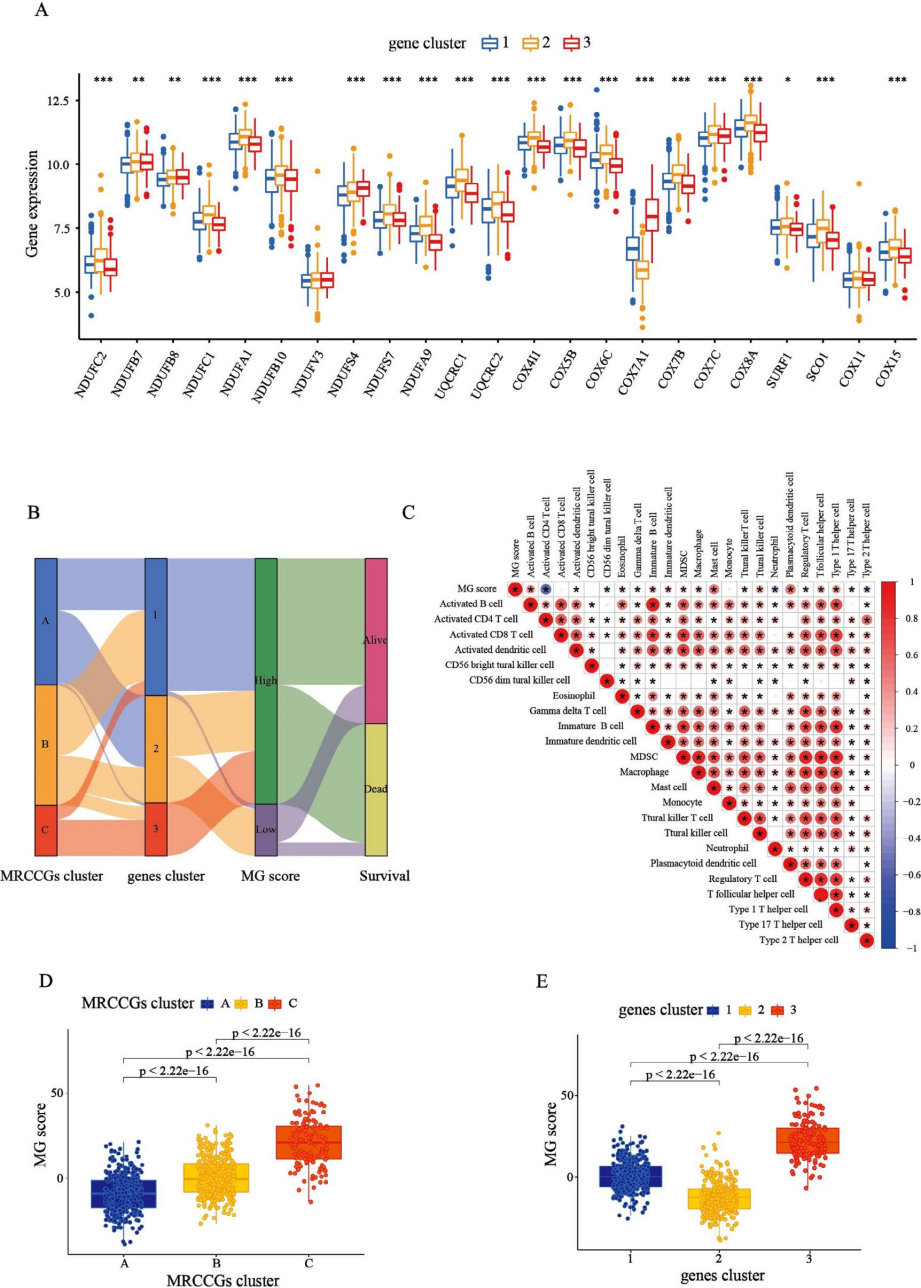
**Identification of the gene subtypes and construction of MG score** **(A)** Differences in the expression of 24 MCCRGs among the three gene subtypes. **(B)** Alluvial diagram of subtype distributions in groups with different MG scores and survival outcomes. **(C)** The correlation analysis between the MG score and immune cells, with red representing positive correlation and blue representing negative correlation. **(D)** Difference analysis of the MG score in the MRCCG patterns. **(E)** Difference analysis of the MG score in the gene subtypes

To further investigate the biological behavior of the three MRCCG patterns identified above, we identified 1175 pattern-related DEGs using the R package “limma” (Fig. 4B) and performed a functional enrichment analysis. GO enrichment analysis (Fig. 4C) showed that the differentially expressed genes were mainly enriched in biological processes including the cell cycle, DNA replication, and mitochondrial translation. The cellular components of the DEGs were significantly enriched in the mitochondrial inner membrane and the mitochondrial matrix. In parallel, these DEGs were mainly involved in the molecular functions of DNA helicase activity and catalytic activity of DNA. KEGG analysis indicated enrichment of the cell cycle and carbon metabolism pathways (Fig. 4D), suggesting that DEGs among the MRCCG subtypes play a vital role in the energy metabolism of the TME. We then conducted a univariate Cox regression analysis to identify the prognostic value of pattern-related DEGs and screened out 555 prognostic DEGs (*p* < 0.05) that were used in the subsequent analysis. A consensus clustering algorithm was used to divide the patients into three gene subtypes based on the prognostic DEGs, namely, gene cluster 1–3 (Fig. S3). The heat map of relationships between clinicopathological features and gene subtypes showed that the expression of most prognostic-related genes was higher in gene cluster 3 and lower in gene cluster 2 (Fig. 4E). In addition, a survival analysis showed that patients in gene cluster 3 had the worst prognosis, whereas patients in cluster 2 had a favorable prognosis (log-rank test, *p* < 0.001; Fig. 4F). Moreover, the three gene cluster subtypes showed significant differences in the expression of MRCCGs, consistent with the results of the three MRCCG patterns (Fig. 5A). Interestingly, TME-related-genes also differentially expressed in the three gene cluster subtypes (Fig. S5).

### Construction of the prognostic MG score

To quantify MRCCG patterns in individual STAD patients, we constructed an MG score model based on subtype-related DEGs. The alluvial diagram comprehensively showed the flow of MG score fraction construction (Fig. 5B). We observed a significant difference in MG scores between MRCCG patterns and gene subtypes. Compared with patterns A and B, MRCCG pattern C had a significantly higher MG score (Fig. 5D). Similarly, the MG score of gene subtype 3 was the highest, whereas that of subtype 2 was the lowest, implying that the high MG score group had a poor prognosis (Fig. 5E). Overall, these results indicate that the three computational methods of classification have a high degree of coincidence. To investigate the potential effect of the MG score on immune regulation of TME, an immune correlation analysis was conducted between the MG score and immune cells. The result showed that the MG score was significantly positively correlated with activated B cells, mast cells, and plasmacytoid dendritic cells, whereas it was negatively correlated with activated CD4^+^ T cells (Fig. 5C).

**Fig. 6:**
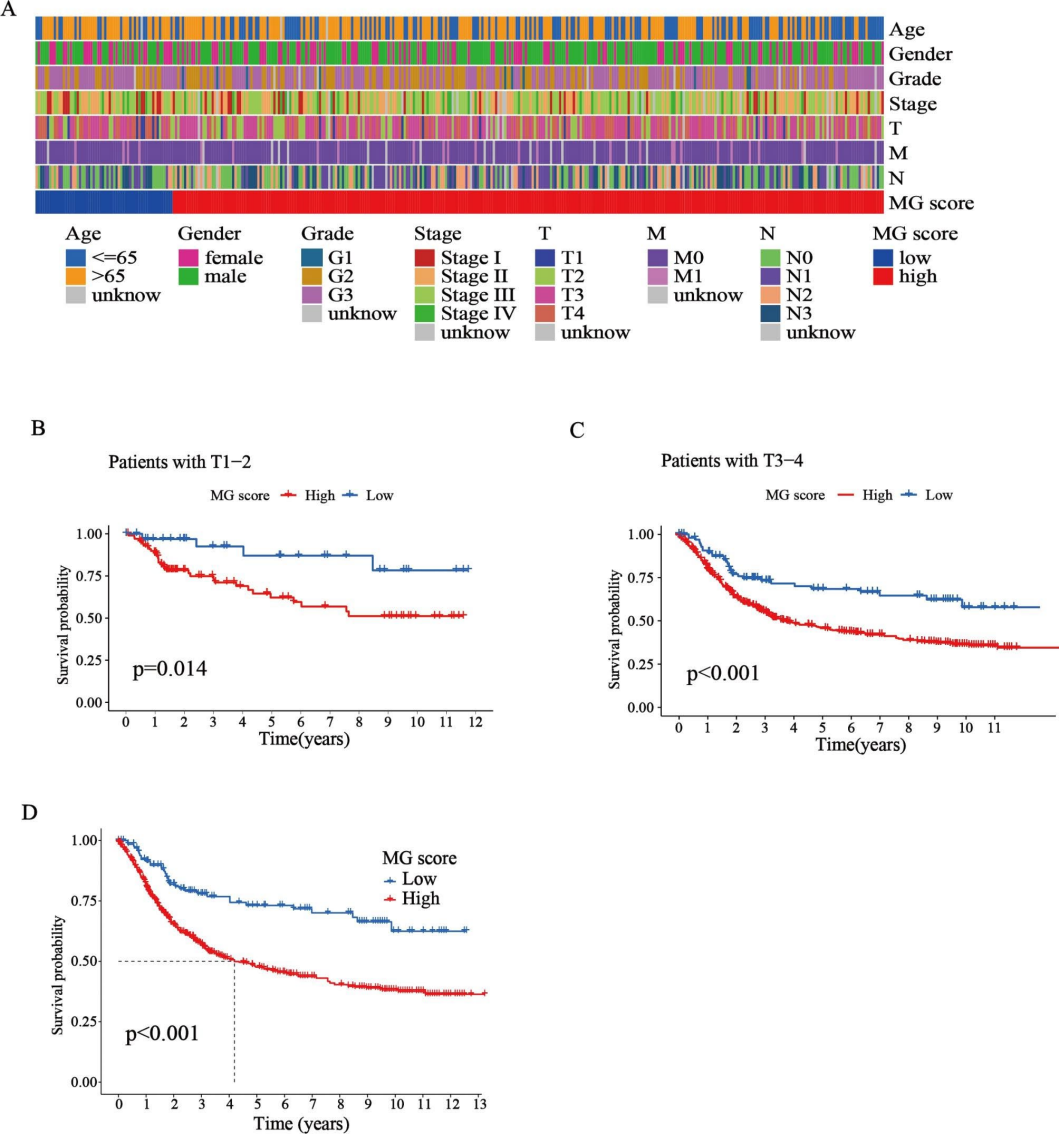
**Identification of the clinical characteristics and predicting the STAD progression** **(A)** Differences in clinicopathologic features of high-and low-MG score groups of STAD from the TCGA cohort. **(B)** Kaplan-Meier analysis of survival rate in patients with T1-2 between high-MG score group and low-MG score group in TCGA cohort. **(C)** Kaplan-Meier analysis of survival rate in patients with T3-4 between high-MG score group and low-MG score group in TCGA cohort. **(D)** Kaplan–Meier analysis of the prognosis between the high-MG score group and low-MG score group (log-rank tests, p-value < 0.001).

To elucidate the effect of the MG score on clinical characteristics, we explored the correlation between the MG score and different clinical features (e.g., age, sex, histological grade, pathological stage, and TNM stage) (Fig. 6A). We observed that the clinical characteristics significantly differed between the high-MG and low- MG score groups. Moreover, the survival analysis in patients with T1–2 or T3–4 stage showed that the prognosis of patients in the high MG score group was poorer than patients in the low MG score group (p < 0.05) (Fig. 6B-C). Kaplan–Meier survival curves revealed that patients with low MG scores had a significantly favorable OS compared to those with high scores (log-rank test, *p* < 0.001; Fig. 6D). Collectively, these results indicate that the MG score can predict the survival probabilities of patients with STAD.

### Evaluation of tumor somatic mutations between the high- and low- MG score groups

**Fig. 7:**
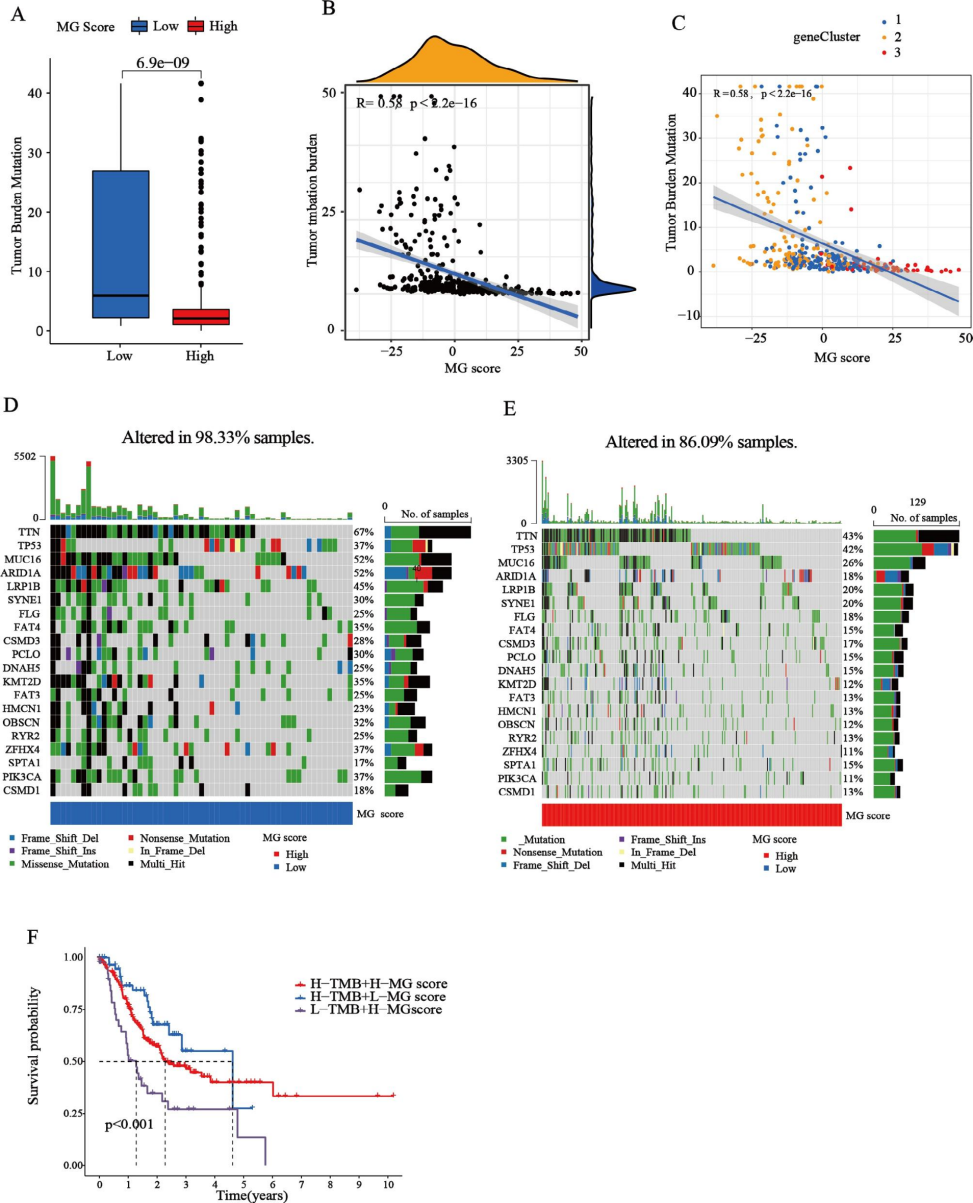
**Evaluation of the MG score and tumor somatic mutation** **(A)** Stratified analysis of the MG score for STAD patients by TMB. **(B)** Spearman correlation analysis of the MG score and TMB. **(C)** Correlations between MG score and TMB calculated by CIBERSORT algorithm. **(D-E)** The waterfall plot of somatic mutation features established with high and low MG scores. Each column represented an individual patient. The upper barplot showed TMB, the number on the right indicated the mutation frequency in each gene. The right barplot showed the proportion of each variant type. (**F**) Survival analysis among three groups of STAD patients who were layered according to both MG-Scores and TMB (log-rank tests, p-value < 0.001). TMB, tumor mutation burden.

Increasing evidence suggests that patients with a high tumor mutation burden (TMB) may benefit from immunotherapy because of the various neoantigens [29]. We determined the optimal cutoff value of TMB (cutoff value = 0.68) by using the minimum p -value method, and divided the patients into a high TMB group (n = 320) and a low TMB group (n = 42). As shown in Fig. 7A, TMB was relatively higher in the low MG score group (Wilcoxon rank-sum test, p = 6.9e-09), indicating that patients in the low MG score group might benefit from immunotherapy. A Spearman correlation analysis demonstrated that the MG score was negatively associated with TMB (R = -0.58, p = 2.2e-16) (Fig. 7B C). As shown in Fig. 7F, when the MG score was integrated with the TMB, the survival curves demonstrated that patients in the low TMB group and the high MG score group had the worst prognosis. We then analyzed the variations in the distribution of somatic mutations between the two MG score groups in STAD patients. The results showed that patients with a low MG score had relatively higher frequencies than those in the high MG score group, with mutation frequencies of 98.33% and 86.09%, respectively (Fig. 7D-E), implying that the low MG score group might benefit from immunotherapy. The top ten mutated genes in the low MG score groups were *TTN*, *MUC16*, *ARID1A*, *LRP1B*, *TP53*, *ZFHX4, PIK3CA, KMT2D*, *FAT4* and *OBSCN* successively. While the top ten mutated genes in the high MG score groups were *TTN, TP53, MUC16, LRP1B, SYNE1, ARID1A, FLG, CSMD3, FAT4* and *PCLO*. Patients with low MG scores had markedly higher frequencies of *TTN*, *MUC16*, and *ARID1A* mutations than those with high MG scores.

### Relationship of MG score with microsatellite instability (MSI) and microsatellite stable (MSS) index

**Fig. 8:**
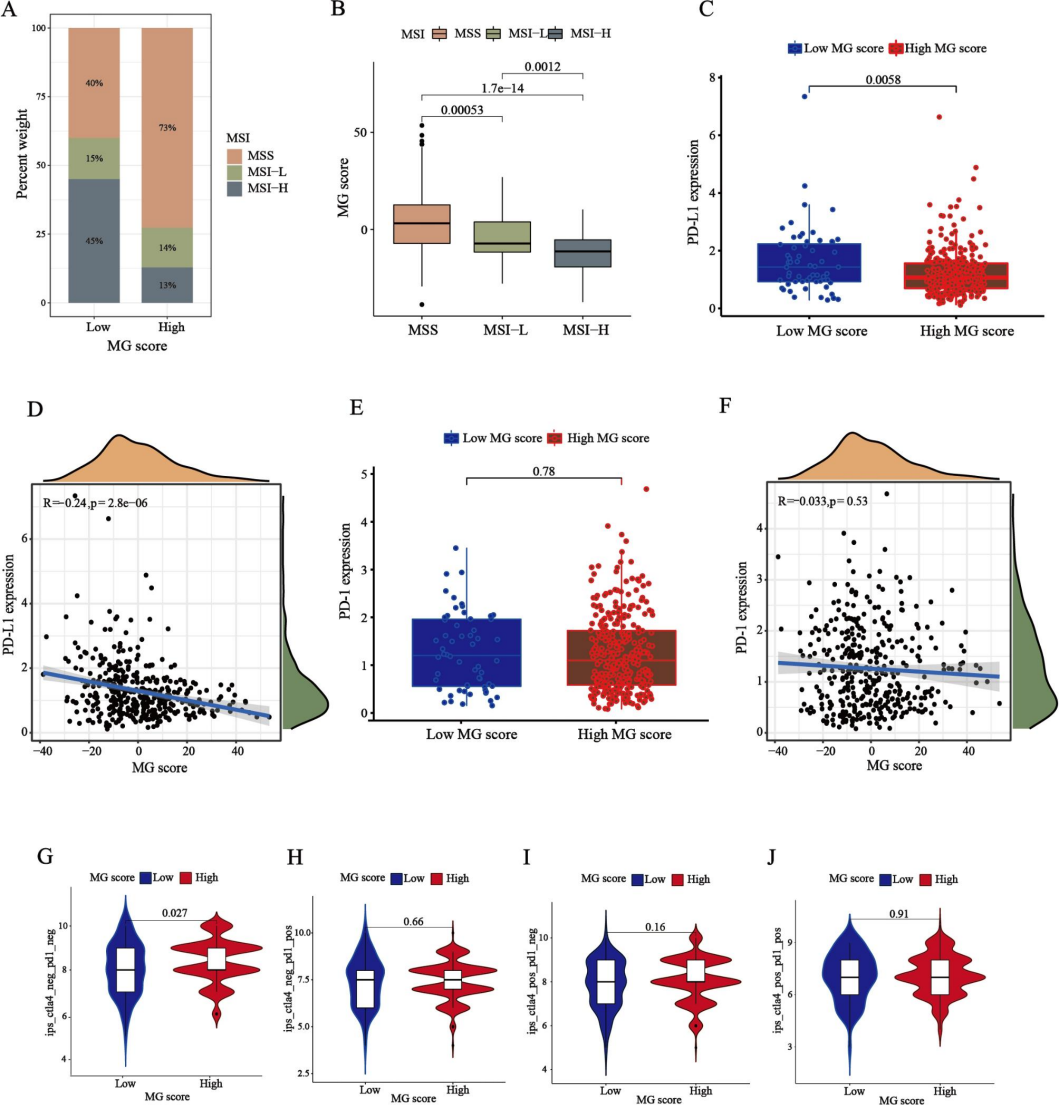
**Comprehensive analysis of the prognostic value according to MG Scores. (A-B)** Relationships between MG score and MSI. **(C)** Stratified analysis of the MG score for STAD patients by PD-L1 **(D)** Correlations between MG score and PD-L1 expression. **(E)** Stratified analysis of the MG scores for STAD patients by PD-1. **(F)** Correlations between MG score and PD-1 expression. **(G)** Differential analysis for low MG score group and high MG score group in CTLA-4 negative and PD-1 negative therapy. **(H)** Differential analysis for low-MG score group and high-MG score group in anti-PD-1 immunotherapy. **(I)** Differential analysis for low MG score group and high MG score group in anti-CTLA-4 immunotherapy. **(J)** Differential analysis for low MG score group and high MG score in anti-PD-1 combined with CTLA-4 immunotherapy. MSI, microsatellite instability; MSS, microsatellite stable.

Mounting evidence suggests that high microsatellite instability (MSI-H) tumors are less responsive to conventional chemotherapy but can benefit from immunotherapy [30, 31]. Correlation analyses revealed that the low MG score group tended to have more MSI-H status, whereas the high MG score group tended to have more MSS status (Fig. 8A-B). Immune checkpoint inhibitors (ICIs) are generally used as therapeutic antitumor agents. However, tumor heterogeneity is a non-negligible factor that potentially limits the efficacy of immunotherapy [32]. Subsequently, we explored the expression of immune checkpoints in the high and low MG score groups. An analysis of immunotherapy scores showed that ICI therapy, represented by the PD-1/PD-L1 inhibitor, played an important role in antitumor therapy. PD-L1 expression was significantly increased in the low MG score group (p = 0.0058) and was negatively associated with the MG score (p = 2.8e-06) (Fig. 8C and D). But PD-1 expression levels and correlations did not show any significance between the high and low MG score groups (Fig. 8E F). Moreover, Fig. 8G J showed that CTLA-4 negative and PD-1 negative therapies had different effects on the high and low MG score groups (*p* = 0.027). These results suggest that the low MG score group was more sensitive to ICI therapy.

### Immune analysis of MG score

**Fig. 9:**
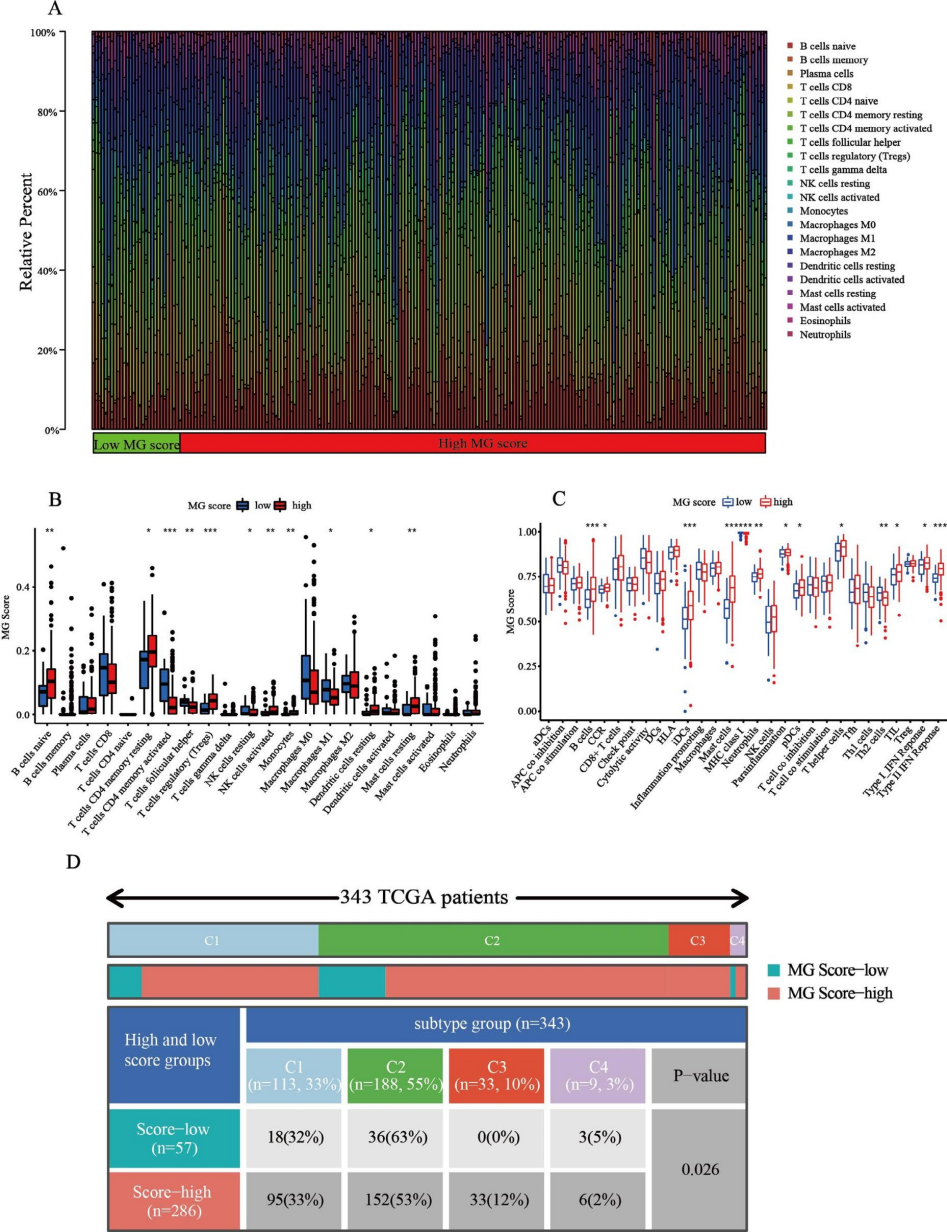
**Comprehensive analysis of immune-related functions between the two MG groups. (A)** Barplot shows the different infiltration abundance of immune infiltrating cells in the two groups. **(B)** The differential expression analysis of immune cells among high MG score and low MG score groups. **(C)** Immune function analysis of high MG score and low MG score groups. **(D)** The difference in four immune subtypes between high MG score and low MG score groups. The *** represents p < 0.001, ** represents p < 0.01, * represents p < 0.05 and ns represents no significance

To investigate the association between the MG score and the abundance of infiltrating immune cells, we performed an immune correlation analysis. We first observed that the distribution of immune cells was noticeably different between the two groups (Fig. 9A). Patients in the high MG score group had a higher enrichment of B cells, CD4 memory resting cells, Tregs, activated NK cells, monocytes, and resting mast cells than patients in the low MG score group, whereas patients in the low MG score group were enriched in activated CD4 memory T cells, follicular helper T cells, resting NK cells, and macrophage M1 (Fig. 9B). In addition, the MG score was closely associated with the different immune functions. High MG scores were closely correlated with B cells, iDCs, mast cells, MHC class I, neutrophils, pDCs, type I IFN response and type II IFN response (Fig. 9C). The therapeutic effect of a tumor is strongly correlated with its immune status, also known as the TME. To further understand the interaction between STAD and its intratumoral immune states, we compared four ‘‘immune subtypes” (C1, C2, C3, and C4) between the high MG score group and low MG score group [33]. C1 (wound healing) is characterized by the high expression of angiogenic genes and a high proliferation rate. C2 (IFN-gamma dominant) has the highest M1/M2 macrophage polarization and shows a high proliferation rate, which may suppress an evolving type I immune response. C3 (inflammatory) is characterized by elevated T_H_17 and T_H_1 genes and lower tumor cell proliferation. C4 (lymphocyte-depleted) displays a more prominent macrophage signature, with T_H_1 suppression and high M2 response [33]. As shown in Fig. 9D, the four immune subtypes were significantly different between the low and high MG score groups (p < 0.05). Notably, the low-MG score group was mainly scattered in C2, implying that this group had higher levels of malignancy.

### MRCCGs is indispensable for ferroptosis

**Fig. 10:**
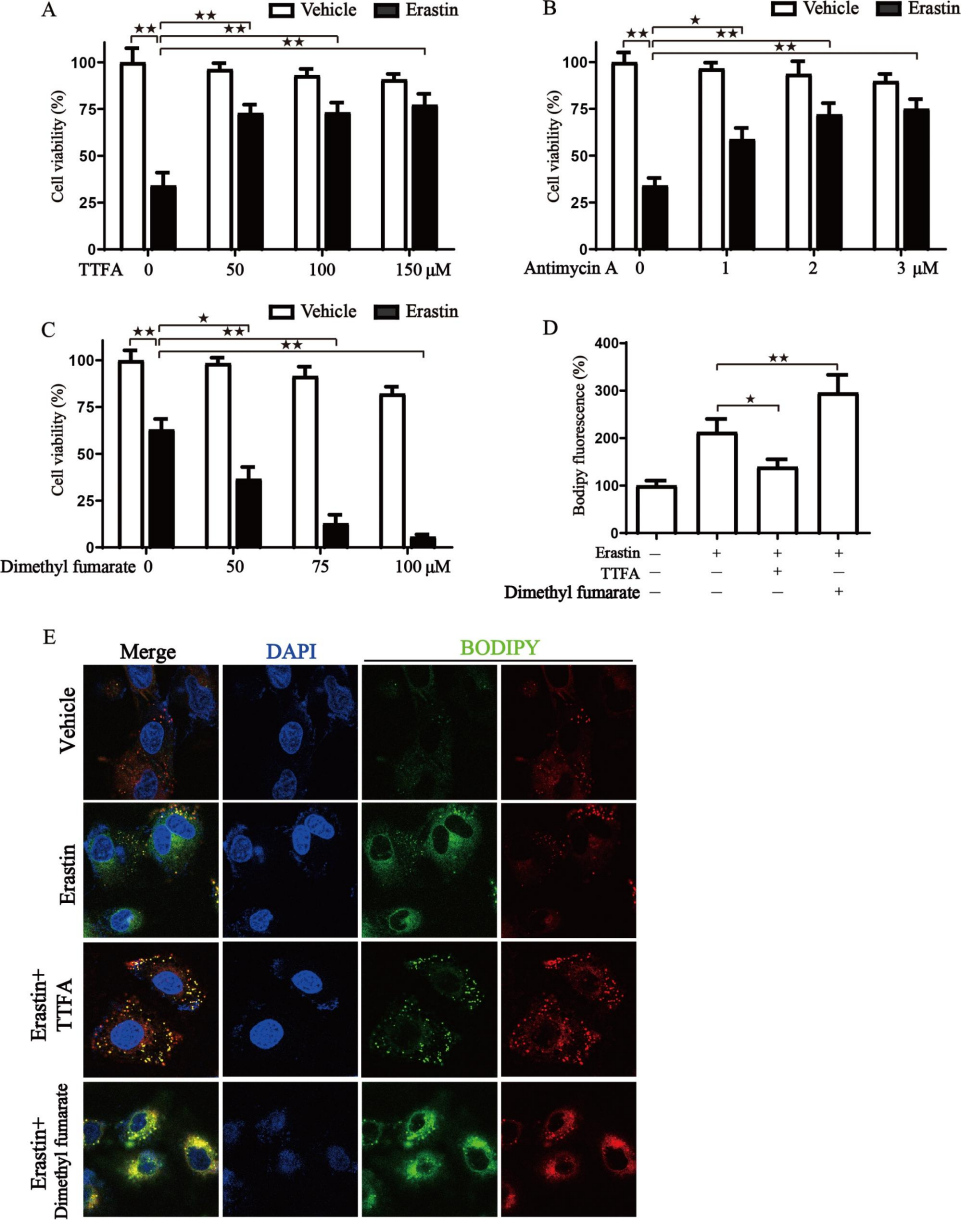
**MRCCGs are indispensable for ferroptosis.** The BGC823 cells were exposed to erastin in the presence or absence of TTFA, Antimycin A, or Dimethyl fumarate, then cell viability was measured by CCK8 assay **(A-C)**, and intracellular lipid ROS labeled with BODIPY was detected by confocal laser microscopy **(D-E)**.

Ferroptosis is a form of regulated cell death that is characterized by iron overload, leading to the accumulation of lethal levels of lipid hydroperoxides [34]. Emerging evidence shows the potential of triggering ferroptosis for cancer therapy [35]. Our previous work has found that achieving ferroptosis via ferroptosis-inducing drugs is effective in many cancers [36–39]. Moreover, our recent studies demonstrated that inhibition of mitochondrial ETC (electron transport chain) attenuates ferroptotic cell death [40]. In this study, heatmaps of expression differences in ferroptosis genes among MRCCGs patterns were presented (Fig S4). The result demonstrated that ferroptosis genes were highly expressed in MRCCGs pattern A and tended to have a better prognosis, which is in line with MRCCGs. Additionally, we wondered whether the MG score could predict the sensitivity to ferroptosis inducing therapy. To validate the above hypothesis, we next employed mitochondrial metabolism inhibitors and mitochondrial energy metabolic substrate in subsequent experiments. The results indicated that cell viability was rescued in BGC823 cells treated with the mitochondrial respiration inhibitors (Fig. 10A-B). However, cell viability was dose-dependently decreased with the addition of mitochondrial metabolism substrate (Fig. 10C). We also detected lipid ROS, which serves as a potent ferroptosis marker. Similarly, accumulated lipid ROS declined in the mitochondrial metabolism inhibitor group, while increased in the mitochondrial metabolism substrate group (Fig. 10D-E). Collectively, these results indicate that MRCCGs play a vital role in ferroptotic cell death induced by erastin.

## Discussion

STAD is a highly heterogeneous disease affected by multiple genetic and environmental factors, which poses a series of challenges to both accurate diagnosis and personalized therapy in STAD [30]. Mitochondrial dysfunction is a common cause of cancer initiation and progression and the mitochondrial electron respiratory chain is often involved in carcinogenesis [31]. Accumulating evidence has shown that single or multiple complexes of the mitochondrial electron respiratory chain play a crucial role in the prognosis of STAD [15, 22, 41]. Moreover, tumor progression relies not only on the proliferation of cancer cells themselves but also on the interaction with the components of the TME [42]. Mitochondrial metabolic, hypoxic, and oxidative stresses are the environmental stress phenotypes in the TME and are considered additional hallmarks of cancer [43]. Determination of the role of MRCCGs in TME cell infiltration is helpful to understand the mechanism of the TME antitumor immune response. Therefore, the combination of TME cell infiltration characteristics in different MRCCGs subtypes will increase the understanding of the TME antitumor immune response of STAD.

In the present study, we revealed global alterations in MRCCGs at the transcriptional and genetic levels in STAD. We classified three distinct molecular patterns based on the expression of 24 MRCCGs using the consensus clustering analysis. The clustering analysis of gene expression profiles is a crucial research topic for cancer subtype diagnosis, which is beneficial for providing more precise treatments for cancer patients [44]. Herein, we applied this efficient method to explore the gene patterns of STAD. Compare to MRCCG pattern B and C, pattern A had better clinicopathological features and a favorable prognosis. Additionally, the STAD MRCCG patterns were also characterized based on significant cellular and energy metabolism, including oxidative phosphorylation, citrate cycle, tricarboxylic acid cycle, and glyoxylate and dicarboxylate metabolism pathways. TME characteristics also significantly differed among the three patterns. Furthermore, we identified three distinct gene subtypes based on DEGs by applying an intersection analysis. Notably, DEGs were mainly involved in energy metabolism and affected the growth and proliferation of tumor cells. Additionally, we found that the clinicopathological characteristics and TME features were also significantly different among the three gene subtypes. Thus, these findings indicated that MRCCGs might serve as a predictor for evaluating the clinical outcome of STAD. More importantly, we established a robust and effective scoring system to quantify MRCCG patterns and validated its predictive ability for the clinical prognosis of patients with STAD. Similarly, the gene subtypes 2 characterized by cellular energy metabolism showed lower MG score, indicating that the MG score may have the ability to evaluate the clinical outcome in STAD. Meaningfully, the low MG score group was correlated with increased MSI-H status, TMB, and PD-L1 expression, indicating they can benefit from immunotherapies. Altogether, this scoring system can be used for prognosis stratification in patients with STAD, will assist in better understanding the molecular mechanism of STAD, and will provide new insight for targeted therapies.

The mitochondrial electron respiratory chain can be considered a target for the treatment of tumors, especially renal cancer [11, 45, 46]. However, some studies discovered that the development of gastric cancer is associated with higher complex I and complex II expressions [15, 16]. To fully understand the underlying mechanism of the mitochondrial electron respiratory chain complex in the occurrence and development of STAD, we investigated 96 genes of the mitochondrial electron respiratory chain complex in STAD and screened out 24 genes which were differentially expressed between STAD and normal tissues. Previous studies have shown that NDUFC2 is associated with a worse prognosis in breast cancer and serves as an acute coronary syndrome biomarker, as well as a target for new therapeutic strategies [47, 48]. Similarly, UQCRC1 is a potential prognostic biomarker and therapeutic target for PDAC. UQCRC1 overexpression results in increased OXPHOS and ATP production, and promotes cell proliferation through the ATP/P2Y2-RTK/AKT axis [49]. Targeting NDUFC1 could be a potential approach in the treatment of gastric cancer. NDUFC1 overexpression was found to be related to more serious tumor infiltration, higher risk of lymphatic metastasis, whereas NDUFC1 downregulation promoted the inhibitory effects on cell proliferation and migration via the PI3K/Akt pathway [50].

Patients with unresectable and/or metastatic gastric cancers generally require systemic therapy. Chemotherapy remains the standard therapy for most patients [51] and immunotherapy has been proven effective in STAD patients with specific molecular subtypes [52, 53]. Recent studies have shown that TME plays a crucial role in STAD tumorigenesis and progression, and outcomes vary according to the different molecular types of STAD [54]. Therefore, obtaining knowledge of the TME helps to understand the immunotherapy response in STAD. To fully understand the relationship between distinct MRCCG patterns and TME cell infiltration characteristics in STAD, we performed immune correlation analyses. A previous study has shown that STAD is characterized by immune cell infiltration containing granulocytes, macrophages, and T lymphocytes [55]. Consistently, we found that the characteristics of TME immune cell infiltration also differed significantly among the three gene subtypes, mainly including activated B cells, activated CD4^+^ T cells, activated CD8^+^ T cells, NK cells, and macrophage cells. These results suggested that the three gene subtypes have a very important significance in shaping different TME landscapes. To further characterize intratumoral immune states of the STAD, we compared four immune signature sets between the high and low MG score groups, which were classified by major immunogenomics methods across 33 cancers analyzed based on the TCGA database [56]. These four categories (C1, C2, C3, and C4) represent characteristics of the TME that largely spurned traditional cancer classifications to create new groups and suggested that specific treatment approaches may be independent of different cancer subtypes [56]. Significantly, C2 (IFN-γ dominant), which is predominantly characterized by a macrophage signature and high proliferation rate, was principally expressed in the low MG score group, with a proportion of 63%. This result indicates that patients in the low MG score group may have a certain connection with macrophages and a higher degree of malignancy than those in the high MG score group. Meaningfully, tumor-associated macrophages are among the most abundant immune cells in the TME [57]. Macrophages promote antitumor responses by killing tumor cells or indirectly recruiting and activating cytotoxic T cells and NK cells in the initial stages of tumor development; they can promote tumor progression, metastasis, and resistance to therapy. Hence, targeting macrophages in cancer cells is a promising therapeutic strategy for cancer [58]. Accordingly, we believe that it is well justified to target macrophages could be a potential treatment for the low MG score group of STAD patients, which requires further validation.

A recent study demonstrated that a high tumor mutation burden (TMB-H) has been proposed as a predictive biomarker for response to immune checkpoint inhibitors (ICIs) [59]. The PD-1 evoked the immune checkpoint response of T cells, resulting in tumor cells capable of evading immune surveillance and being sensitive to immunotherapy [60]. Patients with metastatic cancers probably got a favorable clinical response to ICI, and TMB was used to predict clinical response to ICI in several cancer types [61]. Similar findings suggested that the survival outcomes of patients with H-TMB were correlated with ICI outcomes and had higher responsivity with anti-PD-1, anti-PD-L1, or anti-CTLA4 therapy across diverse solid tumors [62]. In the present study, we observed that TMB and MG score were strongly correlated. In addition, the low MG score group is correlated with higher TMB and had a favorable prognosis. Anti-PD-L1 therapy has recently been used to treat STAD with higher expression of PD-L1 [63]. Similarly, we discovered higher expression levels of PD-L1 in the low MG score group, demonstrating low MG score group may obtain therapeutic effects in anti-PD-L1 treatment. MSI-H has recently been approved by the Food and Drug Administration as a genetic test to select patients for immunotherapy targeting PD-1 and/or CTLA-4 in many cancer types [64]. Currently, MSI-H status and PD-L1 expression are the only established biomarkers associated with the efficacy of certain therapies in patients with advanced-stage gastric and gastroesophageal junction (G/GEJ) cancers [65]. Moreover, a secondary post hoc analysis of the MAGIC trial depicted that compared with patients who had MSI-L tumors, those who had MSI-H tumors had improved survival with surgery alone and inferior survival with perioperative chemotherapy plus surgery [66]. In addition, MSI-high gastric cancer was associated with longer OS and obtained a benefit from ICI therapy while lacking benefit with perioperative or adjuvant chemotherapy. Consistently, the proportion of patients in the low MG score group has a higher percentage of MSI-H status compared to the patients in high MG score group, implying they have a favorable prognosis. Collectively, these results confirm that it’s a good choice for choosing MRCCGs as a predictive biomarker.

Ferroptosis is a newly identified programmed cell death, typically characterized by free iron overload and lethal phospholipid peroxide generation. Increasing evidence has demonstrated that inducing ferroptosis represents a promising therapeutic strategy that preferentially targets iron-rich cancer cells such as HCC [67, 68], NSCLC [69], PDAC [70], leukemia [38] and GC [71, 72], and provides insights into reversing drug resistance in cancers. The mitochondrial electron transport chain is responsible for ATP production. Recent studies provided evidence that mitochondrial electron transport plays indispensable role in the regulation of ferroptosis [73]. In this study, the expression of ferroptosis-related genes was higher in MRCCGs pattern A. Moreover, our *vitro* experiments show that inhibition of electron transport train protects tumor cells against the onset of ferroptosis, while added mitochondrial energy metabolic substrate promotes ferroptosis. These results reveal that MRCCGs-based subtyping and genotyping could be helpful in sensitivity prediction to ferroptosis-based therapy. Recently, targeting MRCCG is evolved as a new therapy to impede the progression of several cancers over the past decades [74]. Therefore, combinational therapy of conventional cytotoxic drugs with MRCCG inhibitor drugs may be an effective regime for combating cancer.

In its clinical and practical applications, our study has its highlights. First, the MG score may be used to assess MRCCGs patterns and corresponding TME cell infiltration characteristics in individual STAD patients to further define the immune phenotype of STAD. Second, the MG score may be used as an independent prognostic biomarker for patients with SATD. Finally, the MG score may predict the efficacy of immunotherapy in STAD patients, allowing for the identification of STAD most likely to benefit from immunotherapy, which providing new insights into individualized treatment of patients with STAD.

## Conclusions

In the present study, we comprehensively elucidate a new scoring system based on 24 MRCCGs by which they affect the TME, clinicopathological characteristics and prognosis. We also determined immunotherapies response and its therapeutic liability for high MG score and low MG score groups. These findings highlighted the significant clinical applications that MRCCGs could be potential biomarkers for STAD and provided a new perspective on developing personalized immune therapeutic strategies for STAD patients.

## Electronic supplementary material

Below is the link to the electronic supplementary material.

Supplementary Material 1

Supplementary Material 2

Supplementary Material 3

Supplementary Material 4

Supplementary Material 5

Supplementary Material 6

Supplementary Material 7

## Data Availability

All data in this study can be obtained from the Gene-Expression Omnibus (GEO; https://www.ncbi.nlm.nih.gov/geo/) and The Cancer Genome Atlas (TCGA; https://cancergenome.nih.gov/abouttcga/).

## List of Abbreviations

STAD: Stomach adenocarcinoma
MRCCs: Mitochondrial respiratory chain complexes
MRCCGs: Mitochondrial respiratory chain complex genes
MSI-H: Microsatellite instability-high
TMB: Tumor mutation burden
TME: Tumor microenvironment
CNV: Copy number variation
GO: Gene ontology
GSVAs: Gene set variation analyses
PCA: Principal component analysis
DEGs: Differentially expressed genes
RFS: Recurrence-free survival
OXPHOS: Oxidative phosphorylation
ROS: Reactive oxygen species
TCGA: The Cancer Genome Atlas
GEO: Gene-Expression Omnibus
ssGSEA: Gene set enrichment analysis
KEGG: Kyoto Encyclopedia of Genes and Genomes
ICIs: Immune checkpoint inhibitors
MSS: Microsatellite stable.
ETC: Electron transport chain
