# Supplementary material for: Identification of mitochondrial respiratory chain signature for predicting prognosis and immunotherapy response in stomach adenocarcinoma
Source: Cancer Cell Int. 2023 Apr 16;23:69. doi: 10.1186/s12935-023-02913-x (PMC10105960; doi:10.1186/s12935-023-02913-x)
Supplement: Supplementary file 1 — Supplementary Material 1 [file 12935_2023_2913_MOESM1_ESM.docx]

Table S1. The whole mitochondrial respiratory chain complex genes

| MRCC I | MRCC II | MRCC III | MRCC IV | MRCC V |
| --- | --- | --- | --- | --- |
| ND1 | SDHA | UQCRC1 | COX1 | ATP5A1 |
| ND2 | SDHB | UQCRC2 | COX2 | ATP5B |
| ND3 | SDHC | UQCRB | COX3 | ATP5C1 |
| ND4 | SDHD | QP-C | COX4I1 | ATP5D |
| ND5 |  | UQCRH | COX5A | ATP5E |
| ND6 |  | UCRC | COX5B | ATP5J |
| ND4L |  | UQCR | COX6A2 | ATP5O |
| NDUFA2 |  | CYTB | COX6A1 | ATP6 |
| NDUFA3 |  | UQCRFS1 | COX6B1 | ATP5F1 |
| NDUFB3 |  |  | COX6C | ATP5G1 |
| NDUFA6 |  |  | COX7A2 | ATP5G2 |
| NDUFA7 |  |  | COX7A3 | ATP5G3 |
| NDUFC2 |  |  | COX7A1 | ATP5H |
| NDUFB4 |  |  | COX7A2L | ATP5I |
| NDUFB6 |  |  | COX7B | ATP5J2 |
| DAP13 |  |  | COX7C | ATP5L |
| NDUFB7 |  |  | COX8A | ATP8 |
| NDUFB9 |  |  | NDUFA4 | ATP5S |
| NDUFB2 |  |  | COX17 | ATPIF1 |
| NDUFB8 |  |  | SURF1 |  |
| NDUFC1 |  |  | SCO1 |  |
| NDUFB1 |  |  | COX11 |  |
| NDUFA1 |  |  | COX15 |  |
| NDUFB10 |  |  |  |  |
| NDUFB5 |  |  |  |  |
| NDUFV3 |  |  |  |  |
| NDUFAB1 |  |  |  |  |
| NDUFS6 |  |  |  |  |
| NDUFA5 |  |  |  |  |
| NDUFS5 |  |  |  |  |
| NDUFS4 |  |  |  |  |
| NDUFA8 |  |  |  |  |
| NDUFS7 |  |  |  |  |
| NDUFS8 |  |  |  |  |
| NDUFV2 |  |  |  |  |
| NDUFS3 |  |  |  |  |
| NDUFA9 |  |  |  |  |
| NDUFA10 |  |  |  |  |
| NDUFS2 |  |  |  |  |
| NDUFV1 |  |  |  |  |
| NDUFS1 |  |  |  |  |
