# Supplementary material for: Identification of mitochondrial respiratory chain signature for predicting prognosis and immunotherapy response in stomach adenocarcinoma
Source: Cancer Cell Int. 2023 Apr 16;23:69. doi: 10.1186/s12935-023-02913-x (PMC10105960; doi:10.1186/s12935-023-02913-x)
Supplement: Supplementary file 2 — Supplementary Material 2 [file 12935_2023_2913_MOESM2_ESM.docx]

| MRCC I | MRCC III | MRCC IV |
| --- | --- | --- |
| NDUFC2 | UQCRC1 | SURF1 |
| NDUFC1 | UQCRC2 | COX6C |
| NDUFA9 |  | COX7A1 |
| NDUFB10 |  | COX4I1 |
| NDUFA1 |  | COX11 |
| NDUFV3 |  | COX5B |
| NDUFS7 |  | COX8A |
| NDUFB7 |  | COX7B |
| NDUFS4 |  | COX15 |
| NDUFB8 |  | SCO1 |
| NDUFA6 |  | COX7C |

Table S2. 24 significantly expressed MRCCGs between STAD and normal tisssues
