# Supplementary figures and images for: Identification of mitochondrial respiratory chain signature for predicting prognosis and immunotherapy response in stomach adenocarcinoma
Source: Cancer Cell Int. 2023 Apr 16;23:69. doi: 10.1186/s12935-023-02913-x (PMC10105960; doi:10.1186/s12935-023-02913-x)

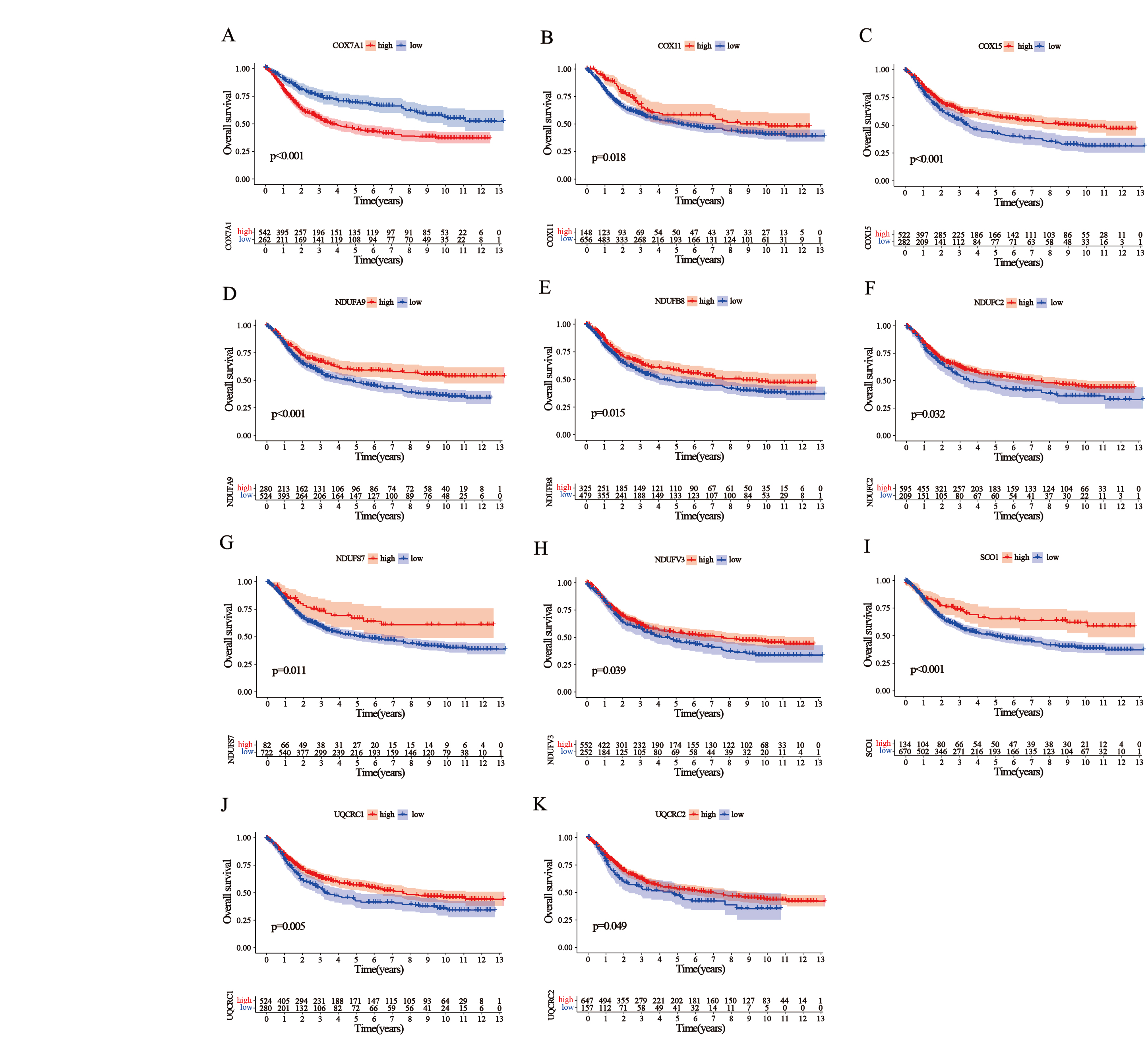

Supplement: Supplementary file 3 — Supplementary Material 3 [file 12935_2023_2913_MOESM3_ESM.jpg]

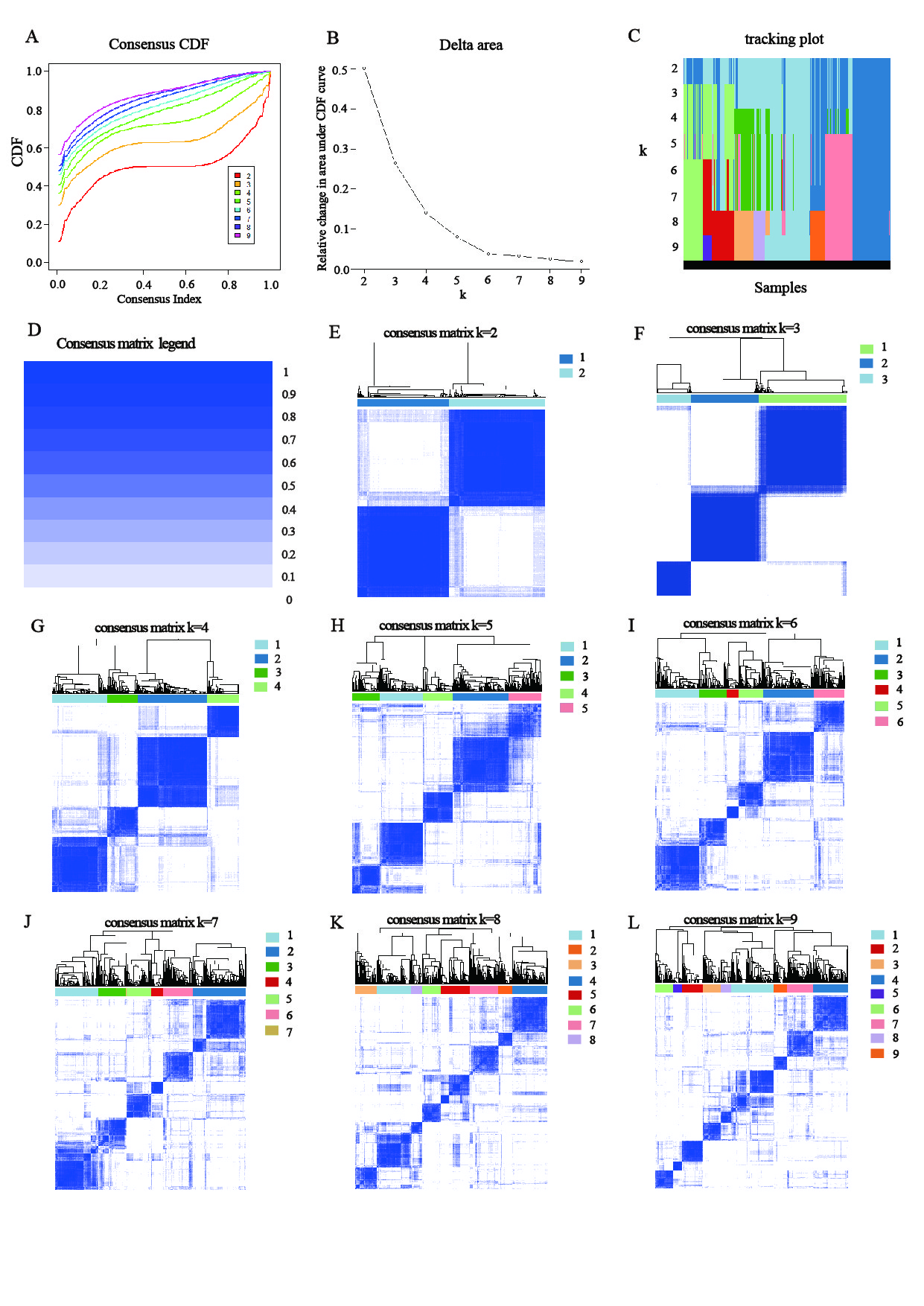

Supplement: Supplementary file 4 — Supplementary Material 4 [file 12935_2023_2913_MOESM4_ESM.jpg]

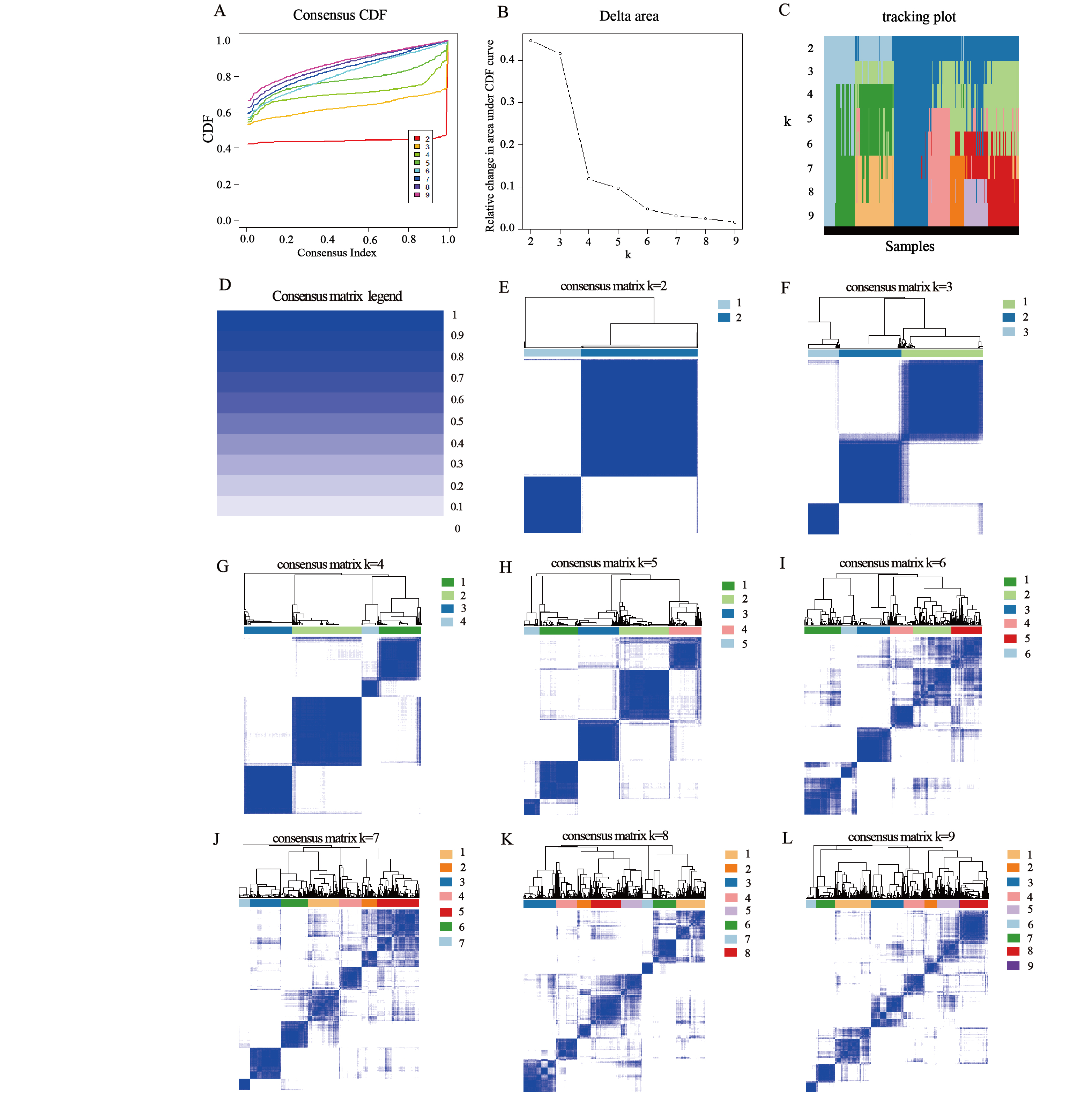

Supplement: Supplementary file 5 — Supplementary Material 5 [file 12935_2023_2913_MOESM5_ESM.jpg]

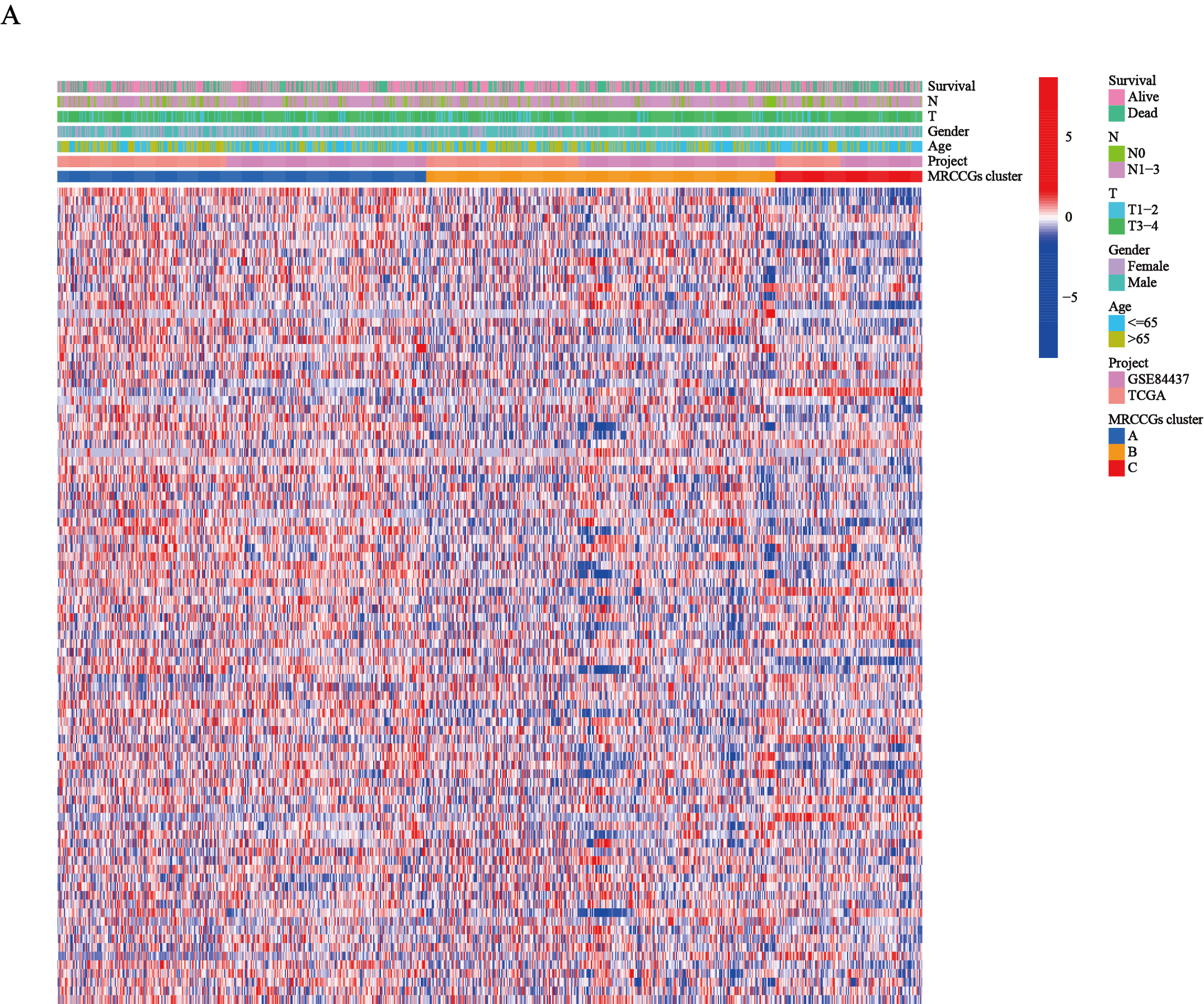

Supplement: Supplementary file 6 — Supplementary Material 6 [file 12935_2023_2913_MOESM6_ESM.jpg]

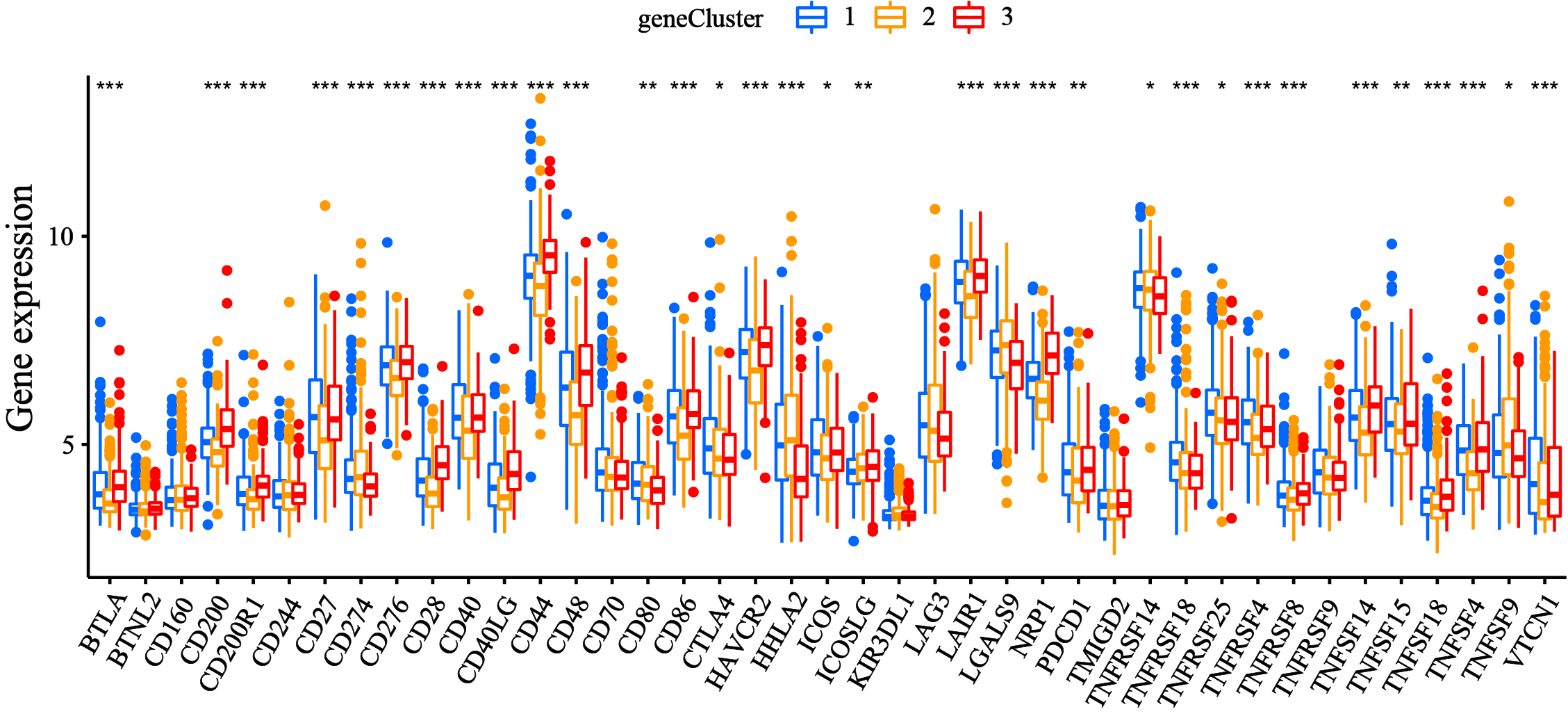

Supplement: Supplementary file 7 — Supplementary Material 7 [file 12935_2023_2913_MOESM7_ESM.jpg]
